# Supplementary material for: Bacillus velezensis Y6, a Potential and Efficient Biocontrol Agent in Control of Rice Sheath Blight Caused by Rhizoctonia solani
Source: Microorganisms. 2024 Aug 16;12(8):1694. doi: 10.3390/microorganisms12081694 (PMC11357648; doi:10.3390/microorganisms12081694)
Supplement: Supplementary file 1 [file microorganisms-12-01694-s001.zip › Supplementary Material.pdf]

Bacillus velezensis Y6, a potential and efficient biocontrol agent in control of rice sheath blight caused by *Rhizoctonia solani*

Huan Tao[1,2†], Xiaoyu Li[2†], Huazhen Huo[1],Yanfei Cai[2*], Aihua Cai[1*]

[1]Guangxi Key Laboratory of Plant Functional Phytochemicals and Sustainable Utilization, Guangxi Institute of Botany, Guangxi Zhuang Autonomous Region and Chinese Academy of Sciences, Guilin 541006, China; taohuanscau@163.com(H.T.); 18978363118@163.com(H.H.)

[2]College of Natural Resources and Environment, South China Agricultural University, Guangzhou 510462, China; taohuanscau@163.com(H.T.); 3216063685@qq.com(X.L.)

* Correspondence: 356542930@qq.com(A.C.);yanfeicai@scau.edu.cn(Y.C.)

†These authors have contributed equally to this work and share the first authorship

Table S1 Strains used in this study

Strain	Genotype	Reference/source
Y6	*B. velezensis* Y6 Wild type	Lab stock
453	*B. velezensis* Y6 *srfAA::mls*	Lab stock
454	*B. velezensis* Y6 *ituA::mls*	Lab stock
459	*B. velezensis* Y6 *fenC::spc*	Lab stock
AG1-IA GD118	*Rhizoctorzia solani*	Lab stock

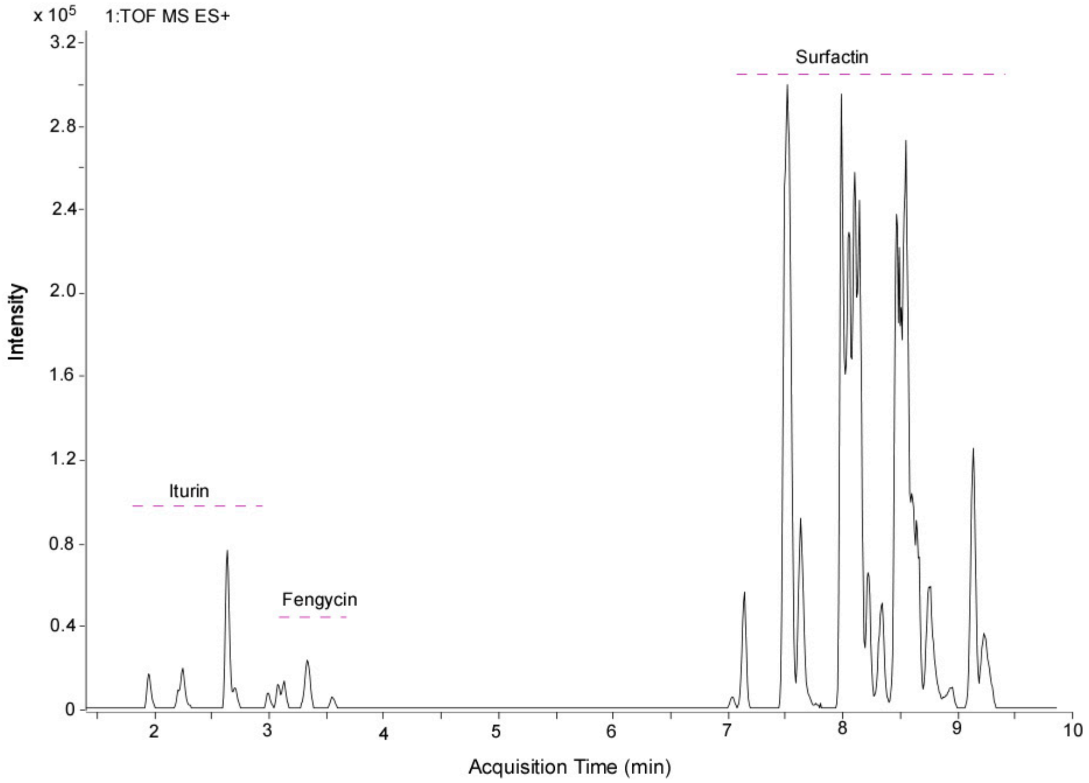

Figure S1. Representative chromatograms of the LPs (iturin, fengycin, and surfactin) from *B. velezensis* Y6

using UPLC/Q-TOF-MS analysis.

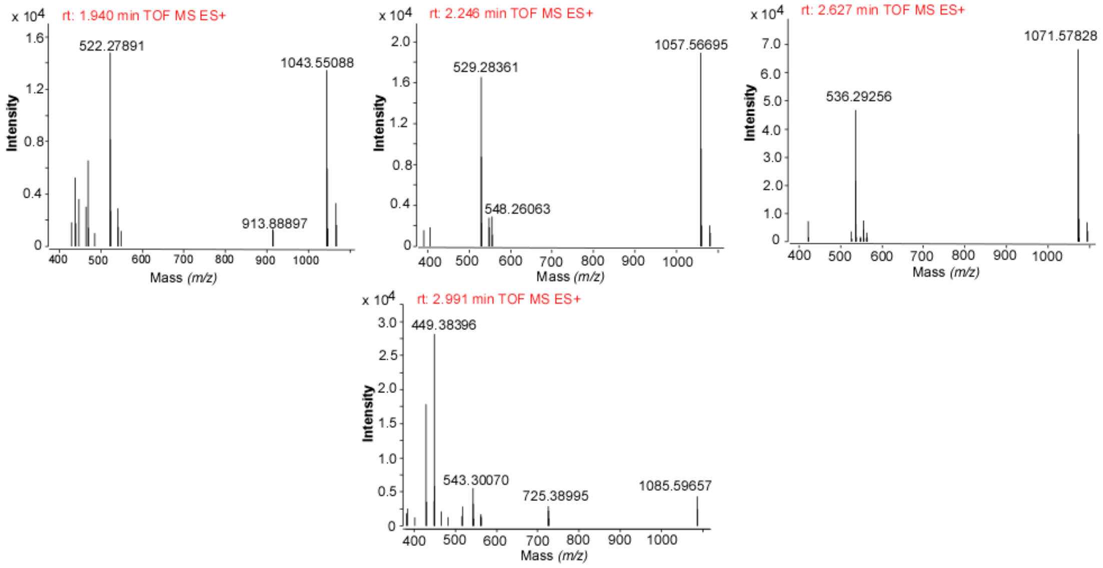

Figure S2. UPLC/Q-TOF analysis of crude extract lipopeptide of *B. velezensis*Y6. [M +H]$^+$ ions of iturin at the m/z of 1043.55088,1057.56695, 1071.57828, and 1085.59657.

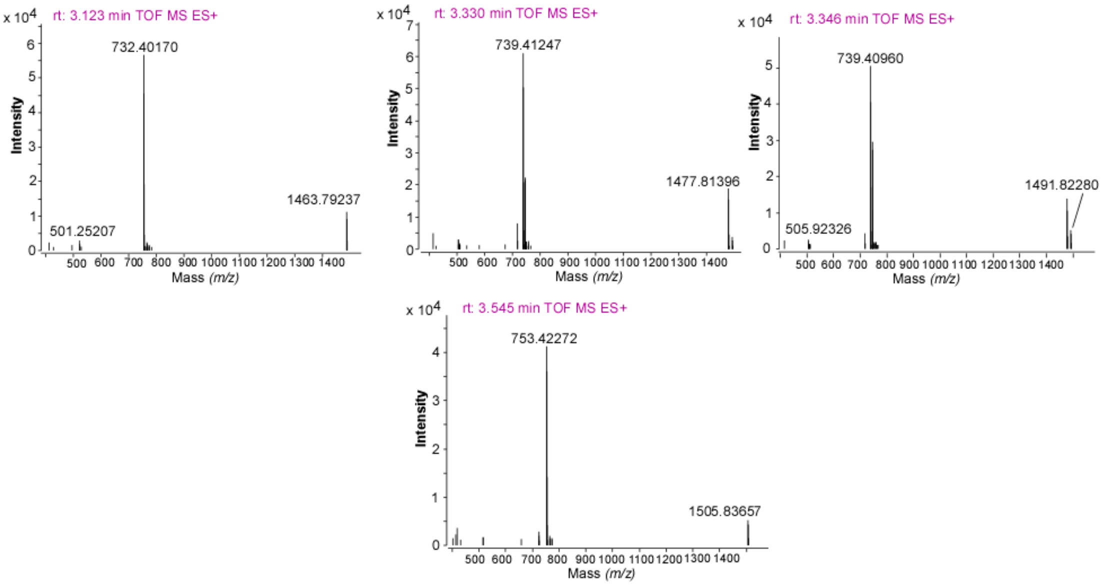

Figure S3. UPLC/Q-TOF analysis of crude extract lipopeptide of *B. velezensis*Y6. [M +H]$^+$ ions of fengycin at the m/z of 1463.79237,1477.81396, 1491.82280, and 1505.83657.

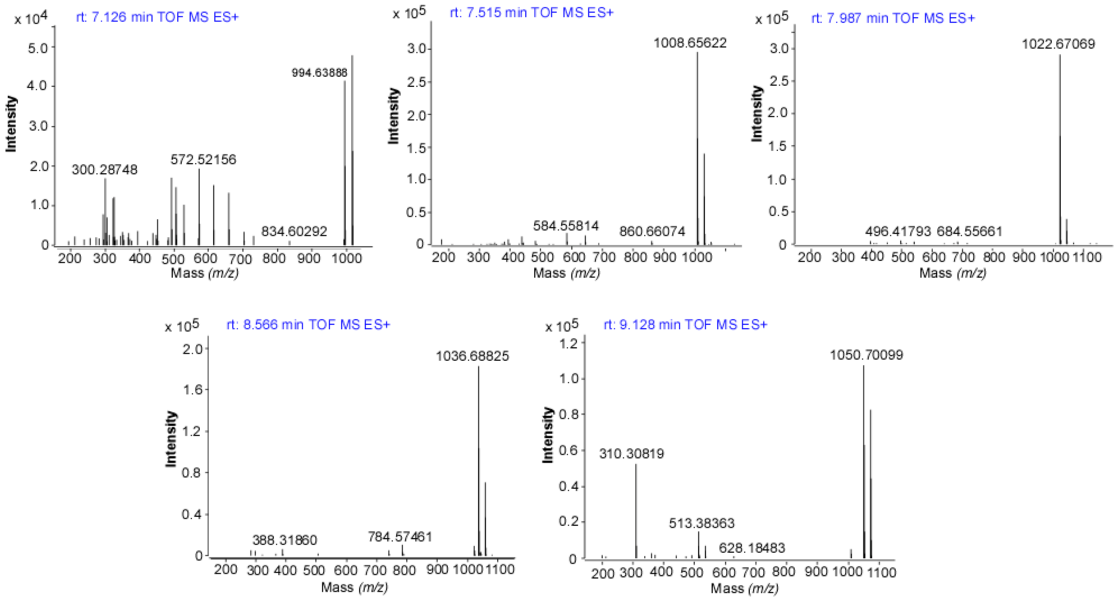

Figure S4. UPLC/Q-TOF analysis of crude extract lipopeptide of *B. velezensis*Y6. [M +H]+ ions of surfactin at the m/z of 994.63888,1008.65622, 1022.67069, 1036.68825, and 1050.70099.

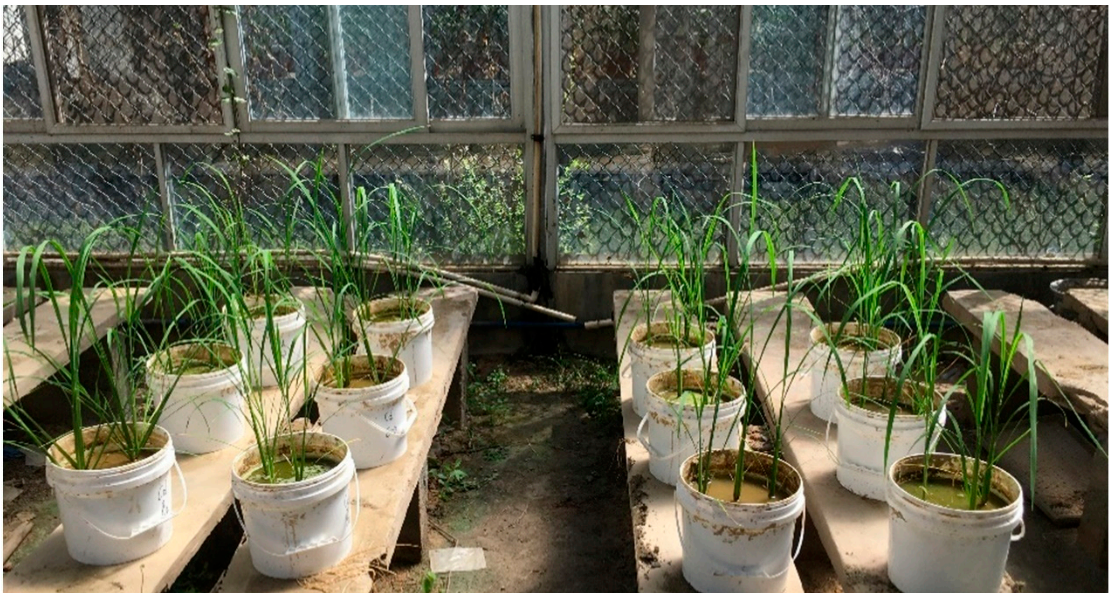

Figure S5. Photographs of a pot experiment on the effect of *B. velezensis*Y6 on rice growth.

Table S2 The effect of *B. velezensis*Y6 on rice growth

	**CK**	**CY18**
Effective panicle number per plant	10.17±1.340a	12.33±0.94b
Thousand-grain weight (g)	18.05±0.21a	19.03±0.37a
Seed-setting rate (%)	66.50±2.06a	81.25±1.48b

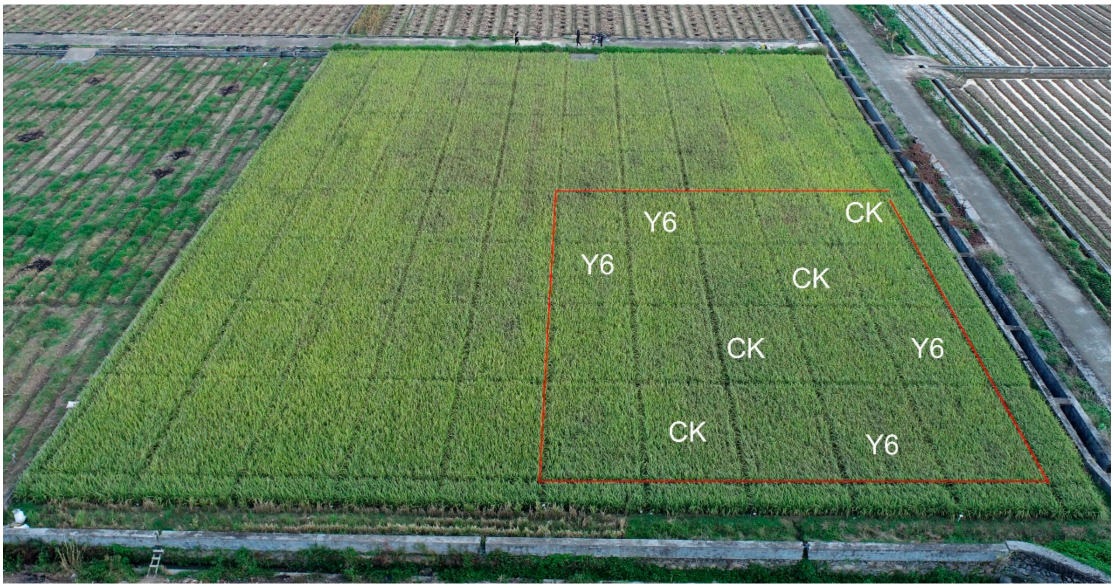

Figure S6. Photographs of field experiment on the effect of *B. velezensis* Y6 on rice yield

Table S3. Effects of *B. velezensis* Y6 on rice yield in field

	CK	Y6
Panicle length (cm)	21.950±1.840a	23.650±2.330b
Thousand-grain weight (g)	17.120±0.510a	18.050±0.510a
Rice yield (dry weight) (kg/m^2)	0.468±0.043a	0.523±0.052a

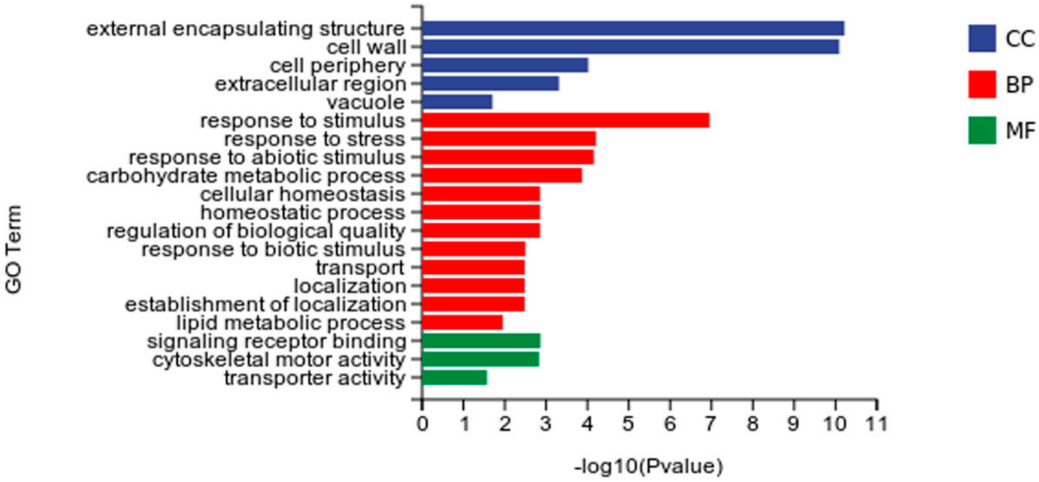

Figure S7. Top 20 pathways with the most significant enrichment in GO term in the transcriptome of rice inoculated with B. velezensis Y6 and control. The X-axis is GO term, indicating the number and percentage of DEGs under each functional classification; the Y-axis is GO term enrichment -log10 (p value), indicating the enriched GO functional classification, which is divided into three categories: cellular components(CC), molecular functions(MF), and biological processes(BP).

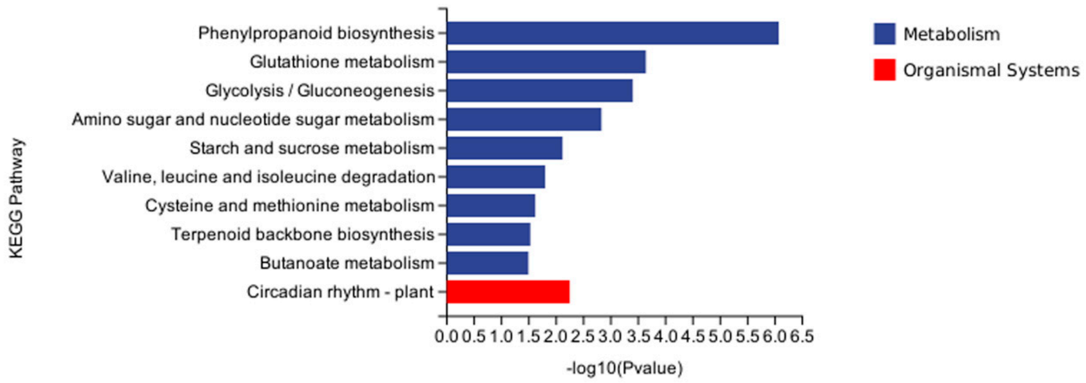

Figure S8. Top 10 pathways with the most significant enrichment in KEGG pathway in the transcriptome of rice inoculated with B. velezensis Y6 and control. The X-axis is pathway, indicating the number and percentage of DEGs under each functional classification; the Y-axis is pathway enrichment -log10 (p value), indicating the enriched KEGG pathway functional classification, which is divided into metabolism and organismal systems.
